# Linking voice pitch to fighting success in male amateur mixed martial arts athletes and boxers

**DOI:** 10.1017/ehs.2021.45

**Published:** 2021-09-10

**Authors:** Christoph Schild, Ingo Zettler

**Affiliations:** 1 Department of Psychology, University of Siegen, Adolf-Reichwein-Str. 2a, 57068 Siegen, Germany; 2 Department of Psychology, University of Copenhagen, Øster Farimagsgade 2a, 1353 Copenhagen, Denmark

**Keywords:** Voice pitch, fundamental frequency, formidability, mixed martial arts, boxing

## Abstract

Whereas voice pitch is strongly linked to people's perceptions in contexts of sexual selection, such as attractiveness and dominance, evidence that links voice pitch to actual behaviour or the formidability of a speaker is sparse and mixed. In this registered report, we investigated how male speakers’ voice pitch is linked to fighting success in a dataset comprising 135 (amateur) mixed martial arts and 189 (amateur) boxing fights. Based on the assumption that voice pitch is an honest signal of formidability, we expected lower voice pitch to be linked to higher fighting success. The results indicated no significant relation between a fighter's voice pitch, as directly measured before a fight, and successive fighting success in both mixed martial arts fighters and boxers.

## Graphical abstract

**Figure:**
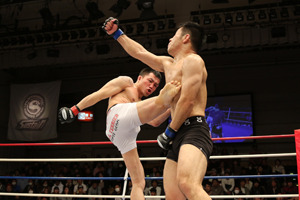

**Social media summary:** We found no relation between voice pitch and fighting success in a dataset comprising 324 boxing and mixed martial arts fights.

## Theoretical background

The human voice plays a crucial role in contexts of sexual selection (e.g. Hodges-Simeon et al., 2010; O'Connor et al., 2014; Puts et al., 2016; Rosenfield et al., 2019), such as mate choice (Pisanski et al., 2018). Herein, especially one vocal characteristic, the fundamental frequency (mean F0), has repeatedly been linked to relevant perceptions and characteristics of speakers. F0 influences perceptions of voice pitch, such that lower F0 is associated with deeper pitch. Within sexes, F0 predicts various criteria across contexts and has been linked to, for example, reproductive success (e.g. Apicella et al., 2007; Rosenfield et al., 2019), hunting reputation (Smith et al., 2017) and corporate hierarchy (Mayew et al., 2013). In male speakers, lower F0 is typically also related to being perceived as more attractive (e.g. Feinberg et al., 2005; Jünger et al., 2018; Puts et al., 2016) and more dominant (e.g. Borkowska & Pawlowski, 2011; Hodges-Simeon et al., 2010; Puts et al., 2016).

Meta-analytic evidence suggests that such perceptions are based on an honest signal of F0, as F0 provides valid information about the physical condition and formidability of speakers. For example, male individuals with lower F0 are taller (Pisanski et al., 2014) and stronger (Aung & Puts, 2019) and have higher testosterone levels (Aung & Puts, 2019). Importantly, however, these relations are relatively weak (*r*_height/F0_ = −0.13, *r*_strength/F0_ = −0.07 and *r*_testosterone/F0_ = −0.20; meta-analytic references above) and – for strength and testosterone – based on a relatively small number of studies (*k*_strength/F0_ = 13, *k*_testosterone/F0_ = 8) with relatively small sample sizes (mean *N*_strength/F0_ = 65.00, mean *N*_testosterone/F0_ = 95.38). Further, strength and height are rather indirect proxies for formidability, whereas evidence that links F0 to actual formidability of an individual is sparse. Although lower F0 predicted better hunting reputation in hunter–gatherers (Smith et al., 2017), studies focusing on intrasexual physical competition are hardly existing and/or contradictory. More precisely, given reduced direct male–male competition in contemporary societies, studies investigating whether traits – e.g. beardedness (Dixson et al., 2018), facial morphometrics (Zilioli et al., 2015) and vocal characteristics (Šebesta et al., 2019) – are linked to success in male–male competition have focused on sporting contests, ‘which remain active Darwinian arenas in which direct same-sex competition for status readily occur’ (Dixson et al., 2018: 148). Herein, mixed martial arts (MMA) are typically considered as a good proxy of contest competition in ancestral human environments (e.g. Dixson et al., 2018), as fights are limited by a few regulations only (e.g. strikes to genitals and eyes are forbidden). Importantly, the best MMA fighters typically receive very high rewards for competing, which suggests that success is indeed linked to higher societal status. Payouts for a single bout in the recent Ultimate Fighting Championship 249 event reached US$500,000 (DAZN, 2020).

While previous research suggests that (masculine) facial cues predict fighting success in male but not female MMA fighters (Little et al., 2015; Palmer-Hague et al., 2016; Zilioli et al., 2015), mixed findings exist regarding links between F0 and fighting ability. F0 was not linked to fighting success, as indicated by the ratio between wins and losses, in a sample of amateur MMA fighters (Šebesta et al., 2019). In contrast, F0 was negatively linked to the number of fights and the number of wins, but not to the percentage of wins, in a sample of professional MMA fighters in an unpublished master's thesis (Goetz, 2015). Importantly, Goetz (2015) also found that the standard deviation of F0 (F0 SD), perceived as monotonicity, was linked to the number of fights and the number of wins, but not to the percentage of wins.

As recently argued by Aung and Puts (2019), further research is needed to investigate relations between F0 and formidability. To provide a well-powered test of a potential relation between F0 and formidability, we ran a study including data for 135 amateur MMA fights and 189 amateur boxing fights. This not only deepens our insights into how F0 is related to actual formidability, but also provides a rather direct test of the relation, given that dyadic fight data is available and it can be tested whether the difference in F0 between fighters can predict the outcome of a fight. We hypothesised that, on average, fighters with lower F0 are more likely to win their fights.

## Methods

As of 4 November 2020, videos of 277 amateur mixed-martial arts fights and 413 amateur boxing fights were publicly available via the Youtube channel STREETBEEFS (https://www.youtube.com/channel/UCCA9jYYLcoteMaqynrIAylA). Importantly, before each fight, both fighters provide a short speech sample in which they briefly introduce themselves (e.g. their name and the city they come from).

### Measures

#### Voice measurement

All recordings were cut such that only the full statement of the respective fighter was audible and then analysed for mean F0, F0 SD and F0 CV using PRAAT software (Boersma & Weenink, 2020). If parts of the statement were drowned out by background noise, they were deleted. To this end, two trained research assistants (blind to the research design and the hypothesis) independently listened to each voice recording and rated whether the statement was fully usable (i.e. at no part of the statement is there loud background noise that makes it hard to understand the fighter), partly usable (i.e. at some parts of the statement there is loud background noise that makes it hard to understand the fighter) or unusable (i.e. there is loud background noise that makes it hard to understand the fighter during the whole statement). In cases of disagreement, the two research assistants jointly listened to the corresponding recording again and decided on a rating. Partly usable files were then cut (i.e. parts with loud background noise were deleted). To foster the accuracy of voice pitch measurement, only recordings that were longer than 2 seconds were included in the analyses. Recordings of both fighters were finally deemed usable for 325 fights (136 MMA, 189 boxing). The script for the acoustic analyses is publicly available on the Open Science Framework (Feinberg, 2018). Standard settings for male voices (pitch floor and ceiling were 75 and 300 Hz, respectively, in accordance with programmers’ recommendations; otherwise default settings) were used. Differences in F0 were calculated by subtracting F0 measurements of both fighters.

#### Fighting success

Each fight was coded in line with the referee's decision. That is, the outcome of the fight was coded as ‘0’ or ‘1’ when fighter 1 or fighter 2 was declared the winner, respectively. In one case the result of a fight was a draw. This fight was excluded from the analyses, leaving a final sample of 135 amateur MMA fights and 189 amateur boxing fights.

#### Control variables for robustness checks

In some cases, the fighting record (e.g. five wins, one loss) of each fighter was mentioned before a fight. Accordingly, we computed a win–loss ratio for each fighter and calculated a win–loss ratio difference for each pair of fighters (before they fought against each other), if applicable. Corresponding analyses were thus based on a smaller sample size. Further, we had two research assistants (the same who rated the usability of the recordings) rate which fighter was taller (0 = fighter 1, 1 = fighter 2). In cases of disagreement, the two research assistants watched the corresponding fight again jointly and decided on a rating. Both variables were used as control variables in additional logistic regressions (i.e. robustness checks).

### Power analysis

Considering that the subjects of our analyses are pairs of fighters (*N* = 135 for MMA fights, *N* = 189 for boxing fights), a sensitivity analysis using G*Power (Faul et al., 2009) suggests that we have power = 0.90 to detect a point-biserial correlation between the difference in F0 and the fight outcome of *r* = 0.24 and *r* = 0.21, respectively (*α* = 0.05, one-tailed). Note that the sensitivity analysis differs from the Stage 1 Protocol. The sensitivity analysis in the Stage 1 Protocol was based on the estimated number of usable datapoints. However, the final sample was smaller than expected.

### Analyses

All analyses were computed with the statistical software R (R Core Team, 2016) and the psych R package (Revelle, 2019). The analysis code and data are publicly available (https://osf.io/rymv5). We first ran separate analyses for MMA and boxing fights. For our main analyses, we conducted point-biserial correlations between the difference in F0 and the fight outcome. One-tailed tests (given the directed hypothesis) and *p*-values were used to make statistical inferences. If *p* was smaller than 0.05, we rejected the null hypothesis. In addition, we ran logistic regressions including F0 difference, type of fight, height difference, and win–loss ratio difference as predictors of the fight outcome. Lastly, we ran exploratory point-biserial correlations between differences in measures of monotonicity (F0 CV and F0 SD) and the fight outcome. Outliers (e.g. very low or high F0) were excluded from the analyses.

## Results

Differences in F0 between fighters were not significantly related to the fight outcome in either MMA fights (*r* = 0.06, *p* = 0.503) or boxing fights (*r* = 0.01, *p* = 0.932). As further summarised in Table 1, differences in F0 were also not significant predictors across different fight types (Models 1 and 2), or when controlling for height (Model 3) and win-rate differences (Model 4).

**Table 1.** Logistic regressions

	Model 1	Model 2	Model 3	Model 4
F0 difference	0.04	0.06	0.03	0.25
	[−0.18, 0.26]	[−0.17, 0.28]	[−0.20, 0.25]	[−0.22, 0.72]
Fight type		0.31	0.28	0.55
		[−0.15, 0.76]	[−0.18, 0.73]	[−0.38, 1.49]
Taller			0.55*	0.40
			[0.10, 1.00]	[−0.51, 1.32]
Difference win-rate				−0.65*
				[−1.16, −0.14]
*N*	324	324	324	96
Pseudo *R*^2^	0.00	0.01	0.03	0.14

All continuous predictors are mean-centred and scaled by 1 standard deviation. *** *p* < 0.001; ** *p* < 0.01; * *p* < 0.05.

Lastly, differences in F0 SD between fighters were not significantly related to the fight outcome in MMA fights (*r* = 0.14, *p* = 0.107) or boxing fights (*r* = −0.03, *p* = 0.770). Similarly, differences in F0 CV between fighters were not significantly related to the fight outcome in MMA fights (*r* = 0.11, *p* = 0.216) or boxing fights (*r* = −0.03, *p* = 0.730).

## Discussion

In a dataset comprising 135 MMA fights and 189 boxing fights we found no significant link between male fighters’ F0 and successive fighting success. That is, whether a fighter's F0, measured directly before a fight, was different from his opponents’ F0 did not allow prediction of the outcome of the fight. These results are in conflict with the assumption that F0 functions as an honest signal of men's formidability (e.g. Aung & Puts, 2019; Puts & Aung, 2019). On the other hand, the results align with prior studies that found no relation between F0 and fighting success in amateur (Šebesta et al., 2019) and professional MMA fighters (Aung et al., 2021). Note that Aung et al. (2021) used an extended version of the dataset used in Goetz (2015), to which we referred to in the introduction. Importantly, though, in Aung et al. (2021), F0 was significantly linked to fighting experience and size of the fighters, and, thus, some components of fighting ability. Consequently, whereas prior studies found that men with lower F0 are taller (Pisanski et al., 2014), stronger (Aung & Puts, 2019), have higher levels of testosterone (Aung & Puts, 2019) and might have greater immunocompetence (Arnocky et al., 2018; Puts et al., 2016; Schild et al., 2020), there is currently no evidence for a direct link between lower F0 and success in actual physical intrasexual competitions.

One potential explanation for these findings is that, in contemporary MMA and boxing fights, fighters are typically matched by weight, which might limit naturally occurring variance. That is, while body size is quite decisive for outcomes of physical competitions across species (e.g. Alcock, 1996; Aung et al., 2021; French & Smith, 2005), current datasets on human physical competitions are typically limited to within-weight class competition. In conclusion, F0 might still signal formability across weight classes, and thus be a signal of components of fighting ability related to size. A second potential explanation for the findings might be that there is indeed no strong relation between F0 and fighting success, but the relation might be rather subtle – if at all – and thus not reliably detectable with the so far considered datasets.

This registered report has a few limitations. First, the vocal recordings were not standardised and contained slight background noise in some cases. On the other hand, vocal parameters tend to be (highly) correlated across recordings even with different contents (e.g. Mahrholz et al., 2018; Schild et al., 2019). Second, no data was available on the fighters’ anthropometrics and demographics, which would have been important to consider. Third, the amateur fights were not in a standardised environment, such that bouts were in different settings either inside or outside. Further, there was no objective classification of the fighters into weight classes (e.g. by weighing the fighters prior to the fight). Fourth, the range of fighting abilities within the sample was certainly rather narrow (as compared with the range across all male adults) because only amateur fighters were included. Fifth, given stricter regulations in boxing fights, MMA fights might display a better proxy of contest competition in ancestral human environments. Lastly, the current investigation included men only as no dataset of sufficient size including other genders was available.

## Conclusion

In line with prior studies (e.g. Aung et al., 2021; Šebesta et al., 2019) we found no direct link between F0 and fighting success in dataset of 135 MMA fights and 189 boxing fights, respectively. As these results stand in contrast to studies that suggest that F0 might function as an honest signal of men's formidability, future studies should set out to further clarify the role of F0 in contexts of sexual selection.

## Research transparency and reproducibility

The analysis code and data are publicly available on the Open Science Framework (https://osf.io/rymv5/).
